# Executive function and boredom in Japanese monolingual, Chinese monolingual, and Japanese–Chinese bilingual children

**DOI:** 10.3389/fpsyg.2025.1579642

**Published:** 2025-11-17

**Authors:** Siyu Zhao, Izumi Uehara

**Affiliations:** 1 Department of Psychology, Ochanomizu University, Tokyo, Japan; 2 Institute for Education and Human Development, Ochanomizu University, Tokyo, Japan

**Keywords:** executive function, boredom proneness, concentration proneness, state boredom, monolingual students, bilingual students

## Abstract

**Introduction:**

This study examined the relationship between executive function (EF) and boredom-related variables among lower-grade elementary school students, as well as potential differences in these relationships among Japanese (JP) monolingual, Chinese (CH) monolingual, and bilingual children.

**Methods:**

A total of 89 first- and second-grade students participated, including 31 JP monolinguals, 32 CH monolinguals, and 26 JP–CH bilinguals. Participants completed nonverbal and verbal EF tasks, after which their parents assessed their levels of boredom during the tasks, as well as their daily boredom proneness and ease of concentration.

**Results:**

Lower cognitive flexibility was associated with higher daily boredom proneness, regardless of demographic attributes. Among JP monolingual and bilingual children, although the multiple regression model predicting concentration was only marginally significant, girls and those with higher scores on the Japanese word reversal task, an indicator of working memory, tended to show greater ease of daily concentration. Boys were also found to be more prone to boredom during tasks.

**Conclusions:**

These findings suggest that students’ EF abilities, particularly cognitive flexibility and, to a lesser extent, working memory, are more strongly related to daily dispositional tendencies toward boredom and concentration than to temporary boredom experienced during activities.

## 1 Introduction

Boredom is defined as “the aversive experience of wanting, but being unable, to engage in satisfying activity” (Eastwood et al., 2012, p. 482). It has been suggested that boredom encompasses a range of cognitive and emotional processes, including deficits in attention and self-control, diminished meaning and motivation, feelings of emptiness, and a desire for change. However, the relationships among these components remain unclear (Bench and Lench, 2013; Danckert and Merrifield, 2018; Eastwood et al., 2012).

Boredom is typically categorized into two forms: state boredom and boredom proneness (Vodanovich and Watt, 2016). *State boredom* refers to a transient feeling that arises in monotonous situations, such as attending an unengaging lecture or performing repetitive tasks. In contrast, *boredom proneness* represents a dispositional tendency to experience boredom more frequently and intensely in daily life (Tam et al., 2021). While transient experiences of boredom are unlikely to result in major mental health concerns, chronic and intense boredom—reflecting high boredom proneness—has been associated with greater engagement in problematic and dependent behaviors (Blaszczynski et al., 1990; Haager et al., 2018; LePera, 2011; Sommers and Vodanovich, 2000). Nonetheless, the underlying mechanisms of boredom—such as how state boredom emerges, how it affects behavior, and why strong boredom proneness is linked to maladaptive behaviors—remain poorly understood.

It is widely acknowledged that children are more susceptible to boredom than adults; however, empirical research on childhood boredom remains relatively limited (Danckert and Eastwood, 2020). Although some studies have examined the relationship between state boredom in classroom settings and academic achievement, as well as strategies to alleviate boredom in school environments (Jie et al., 2022; Tze et al., 2016), most existing research on childhood boredom has been confined to educational contexts. A deeper understanding of the mechanisms underlying children’s susceptibility to boredom, as well as effective coping strategies, may provide important insights into preventing boredom-related maladaptive behaviors in adulthood (Uehara and Ikegaya, 2024).

This study aims to explore factors that influence children’s state boredom, boredom proneness, and ease of concentration, with the latter trait considered the inverse of boredom proneness (Farmer and Sundberg, 1986; Fisher, 1993; Uehara and Ikegaya, 2025). As a first step, we focus on *executive function* (EF) as a potential factor involved in boredom regulation. Understanding how EF relates to boredom is theoretically important, because boredom has been closely linked to attentional control and self-regulation. Prior research has suggested associations between boredom and the executive control network (Danckert and Merrifield, 2018), as well as between boredom and self-regulatory capacities (Struk et al., 2016). Thus, elucidating this relationship may provide insight into the development of children’s motivation and cognitive engagement, which depend on self-regulatory and attentional control mechanisms. EF is broadly defined as a set of cognitive processes that enable individuals to optimize performance in situations requiring complex cognitive operations (Ward, 2019, p. 398). EF is generally conceptualized as comprising multiple components. Miyake et al. (2000) identified three core components of EF: updating and monitoring of working memory representations (*updating*), inhibition of prepotent responses (*inhibition*), and shifting between tasks or mental sets (*shifting*). Similarly, Diamond (2013) proposed three key EF components: inhibition (including selective attention and cognitive inhibition), working memory, and cognitive flexibility (mental set shifting). Given the conceptual overlap between updating and working memory, as well as between shifting and cognitive flexibility, these constructs can be regarded as closely related.

EF is also conceptually linked to self-regulation, including emotional regulation (Neubeck et al., 2022), and has been shown to be associated with academic performance in children (Bull et al., 2008; Fuhs et al., 2014). However, the relationship between EF and academic performance is complex, likely due to the multifaceted nature of EF. Indeed, research suggests that cognitive flexibility and working memory serve as mediating factors in the relationship between socioeconomic status and academic achievement (Poon et al., 2022).

Several studies have also suggested an association between EF and boredom in adults. Vogel and Fenske (2024) reported that difficulties in emotion regulation were strongly and positively correlated with boredom proneness, and that this association was significantly mediated by attentional difficulties and lower cognitive flexibility. Carriere et al. (2008) demonstrated that the relationship between attention-related cognitive errors and boredom proneness was fully mediated by failures of attention and memory. Gerritsen et al. (2014) argued that these findings point to a link between boredom proneness and specific aspects of EF. In their study, which included scales measuring inattention, hyperactivity, impulsivity, boredom proneness, and executive dysfunction, only executive dysfunction showed a significant regression coefficient predicting boredom proneness. However, these studies assessed EF in adults using self-report measures rather than experimental tasks (Carriere et al., 2008; Gerritsen et al., 2014; Vogel and Fenske, 2024). Thus, the mechanism underlying the relationship between EF and boredom, as well as the developmental emergence of this relationship, remain unclear.

Although the links between EF and boredom have received increasing attention, far less is known about how linguistic and cultural backgrounds relate to boredom. Only a handful of studies have directly compared boredom across cultural groups. For instance, Sundberg et al. (1991) reported that Lebanese and Hong Kong university students scored higher on the Boredom Proneness Scale (BPS) than their Australian and American counterparts, while Vodanovich et al. (2011) found that American students scored significantly higher than German students. Regarding state boredom, Ng et al. (2015) examined the cross-cultural validity of the Multidimensional State Boredom Scale (MSBS) and showed that European Canadians reported higher levels of state boredom than Chinese participants once culturally non-invariant items were excluded. However, because state boredom is highly context-dependent and culturally situated in both its measurement and interpretation, cultural differences in state boredom are difficult to establish. Taken together, existing findings provide inconsistent evidence regarding cultural or linguistic variations in boredom proneness, and almost nothing is known about how bilingualism may relate to children’s experience of boredom.

The present study involves 1st- and 2nd-grade elementary school students, including Japanese (JP) monolinguals, Chinese (CH) monolinguals, and JP–CH bilinguals. Although the primary focus was on the relationship between EF and boredom in children, demographic factors such as gender and language background (monolingual vs. bilingual) were also considered as potential influences on boredom. Prior findings regarding gender differences in boredom are mixed. In Japan, no significant gender differences have been reported in parental ratings of children’s everyday boredom proneness (Uehara and Ikegaya, 2025) or in Boredom Proneness Scale scores among fathers and mothers (Uehara et al., 2024). By contrast, international research has more often suggested that men report higher boredom levels than women: males scored higher on the BPS in U.S. and Australian samples (Sundberg et al., 1991; Vodanovich et al., 2011), and higher state boredom scores were also found among men in a large-scale Chinese survey (Wang et al., 2022). Nevertheless, the evidence remains inconsistent, particularly across cultural contexts. With respect to EF, consistent gender differences have likewise been difficult to detect across studies employing diverse tasks and measures, although Gaillard et al. (2021) highlighted possible sex-related differences in the neural networks supporting executive control. In addition, no prior studies have examined whether bilingualism influences boredom. Findings on bilingualism and EF have also been inconclusive, with several studies failing to demonstrate reliable differences in EF performance between monolingual and bilingual adults (e.g., Dick et al., 2019; Lehtonen et al., 2018; Paap and Greenberg, 2013). These inconsistencies underscore the importance of empirically investigating both gender and language background in relation to boredom during childhood.

Based on the findings from studies conducted primarily in adults, with relatively few studies in children, our preliminary predictions for this study were as follows. Given that no consistent gender differences have been found in certain measures of boredom, we hypothesized that gender differences in boredom would be minimal in this study, with any differences potentially limited to specific boredom measures. Due to the lack of consistent evidence for EF differences between monolinguals and bilinguals in previous research, we anticipated that no significant differences in EF between these groups would emerge in this study either. Given the previously observed association between EF and boredom in adults, we expected that a similar relationship might be observed in elementary school children. This study was unique in its use of three nonverbal and two verbal EF tasks, rather than self-report scales, to more precisely examine the relationship between the three components of EF and boredom. Identifying such associations could help clarify which specific aspects of EF are most relevant to the experience of boredom.

## 2 Methods

### 2.1 Participants

A total of 89 first- and second-grade elementary school students participated in the study. Data for a few children were incomplete due to missing responses (e.g., no correct answers on one of the EF tasks or missing responses on a boredom-related scale). However, because all other data for these participants were available, they were included in the analyses, with the exact sample size reported in each analysis. The final sample consisted of 89 children divided into three groups: a JP monolingual group [31 children (13 boys); *M* age = 6.48 years, *SD* = 0.68, age range = 6–8 years], a CH monolingual group [32 children (19 boys); *M* age = 7.38 years, *SD* = 0.71, age range = 6–8 years], and a JP–CH bilingual group [26 children (13 boys); *M* age = 6.88 years, *SD* = 0.82, age range = 6–8 years]. Due to school privacy regulations, children were not required to disclose their exact birthdate; instead, they provided their school grade and age.

Bilingual children were identified based on parental reports confirming that their child could speak and write both Japanese and Chinese without difficulty, following the identification criteria used in previous research (Weinreich, 1967). The JP monolingual and JP–CH bilingual children resided in suburban areas of Tokyo, Japan, whereas the CH monolingual children lived in Tianjin, China. All participants attended public elementary schools in their respective cities, and their socioeconomic status was classified as middle class. Gender information was obtained through self-report.

### 2.2 Materials and procedures

Before the EF tasks were administered, a preliminary vocabulary assessment was conducted to evaluate children’s verbal ability. The EF battery included three nonverbal tasks—the dot matrix reversal task (Blom et al., 2017), the Simon task (Ross and Melinger, 2017), and the developmental dimensional change card sort (DCCS) task—and two verbal tasks, the digit span task and the word reversal task.

To ensure accurate assessment of responses, all task sessions were videotaped with prior consent from both the children and their parents. After the EF tasks, one parent of each child rated the child’s state boredom (extent of boredom experienced during the tasks), boredom proneness (susceptibility to boredom in daily life), and concentration proneness (tendency to concentrate in daily life). Bilingual children completed both the Japanese and Chinese versions of the two verbal EF tasks.

All study procedures were approved by the Humanities and Social Sciences Research Ethics Committee of Ochanomizu University (approval numbers: 2020–53 and 2020–89). Informed consent was obtained from all participating children and their parents prior to participation. The study was conducted in accordance with the principles of the Declaration of Helsinki.

#### 2.2.1 Preliminary vocabulary assessment

There is no standardized verbal assessment that enables direct comparison of verbal abilities between Japanese and Chinese elementary school students, nor is there a widely accepted vocabulary test for young Chinese learners. To evaluate Japanese vocabulary development, the Picture Vocabulary Test–Revised (PVT-R; Ueno et al., 2008) was administered to JP monolingual and JP–CH bilingual children. The results showed that bilingual children aged 6 and 8 years had significantly lower PVT-R scores than their monolingual counterparts [6-year-olds: *F* (1, 45) = 6.42, η_p_^2^ = 0.125, *p* = 0.015; 8-year-olds: *F* (1, 45) = 5.78, η_p_^2^ = 0.125, *p* = 0.020]. However, no significant differences were found between bilinguals and monolinguals in verbal EF task performance, nor were any significant correlations observed between PVT-R scores and boredom-related variables (*p* > 0.05). Consequently, PVT-R scores were not included in subsequent analyses.

To assess Chinese vocabulary in CH monolingual and JP–CH bilingual children, the PVT-R was translated into Chinese in collaboration with Chinese and Japanese psychologists, under the authorization of Ueno et al. (2008) and Nihon Bunka Kagaku-sha (the copyright holder). This Chinese version was approved for limited use as a reference for evaluating children’s vocabulary levels, but not for direct comparison with the original Japanese version. Even within this reference framework, the Chinese PVT-R scores of bilingual children were significantly lower than those of CH monolinguals [*F* (1, 46) = 18.26, η_p_^2^ = 0.284, *p* < 0.001]. However, as with the Japanese version, there were no significant differences in verbal EF task performance between bilingual and CH monolingual children, nor were any significant correlations found between Chinese PVT-R scores and boredom-related variables (*p* > 0.05). Thus, Chinese vocabulary proficiency was also not considered a significant factor in the present analyses.

#### 2.2.2 EF tasks

Each child’s EF was assessed using five tasks. The nonverbal EF tasks were administered on a customized personal computer (Mouse m-Book K700SN-M2SH2), with stimuli presented on the monitor. Children responded by either pointing to a location on the screen or by pressing the corresponding keyboard key. The verbal EF tasks required spoken responses. JP and CH monolingual children completed each task once, whereas JP–CH bilingual children performed the nonverbal EF tasks once and both the Japanese and Chinese versions of the verbal EF tasks—the Japanese version first, followed by the Chinese version one week later.

#### Dot matrix reversal task

**Figure 1:**
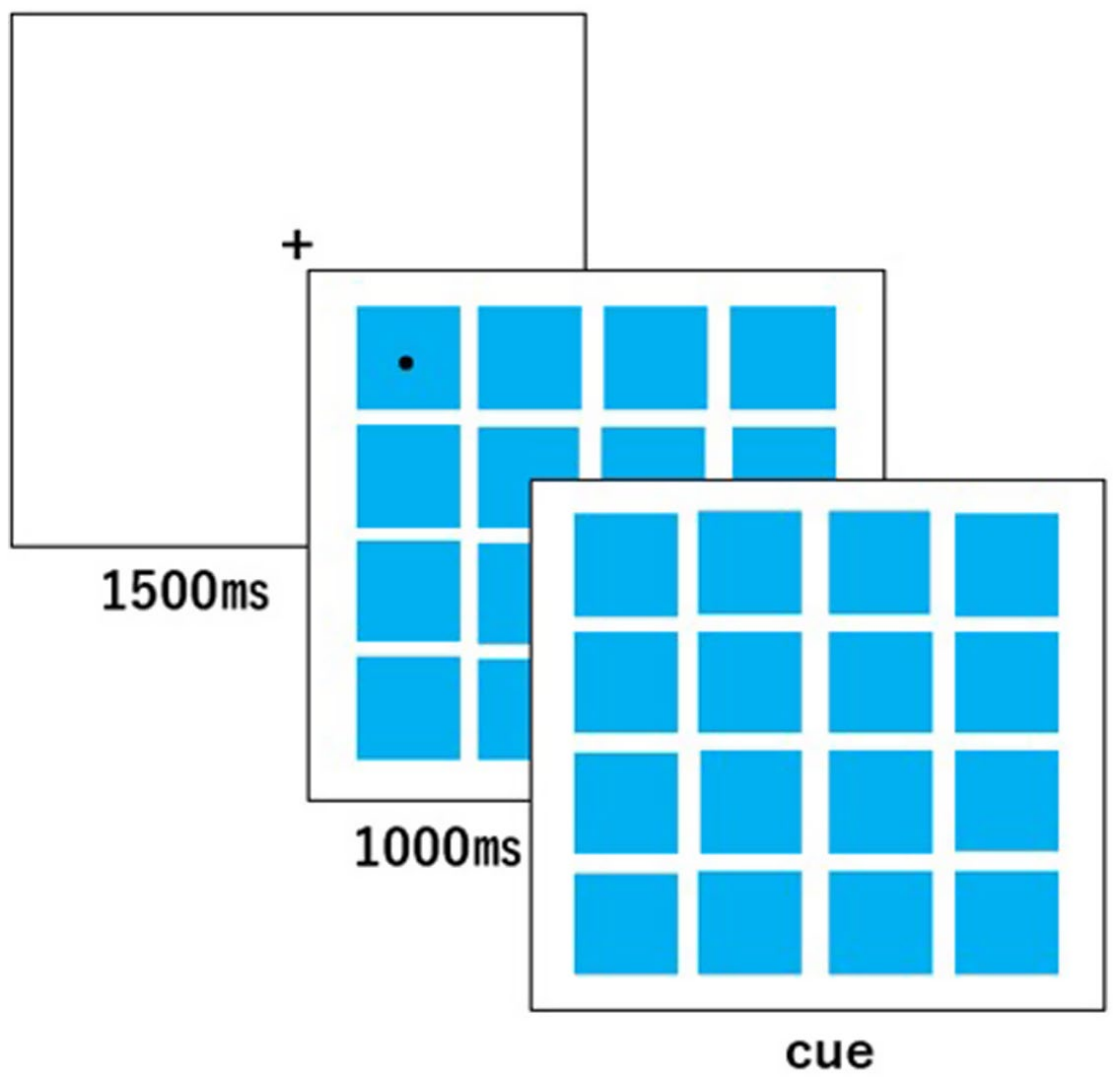
Stimulus presentation in the dot matrix reversal task.

The dot matrix reversal task (Blom et al., 2017; Figure 1) was used to assess working memory. In this task, the children viewed dots appearing one by one on a 4 × 4 grid displayed on the monitor. Immediately after the sequence was presented, they were required to point to the dots in reverse order. The task consisted of six blocks, each containing six trials. In Block 1, a single dot appeared; in Block 2, two dots appeared sequentially. The number of dots increased progressively with each block, up to six dots in Block 6. The locations and presentation order of the dots were automatically and pseudo-randomly determined by the experimental program. Each child’s task score was calculated as the total number of trials in which the dots were correctly indicated in reverse order, yielding a possible range of 0–36 points.

#### Simon task

**Figure 2:**
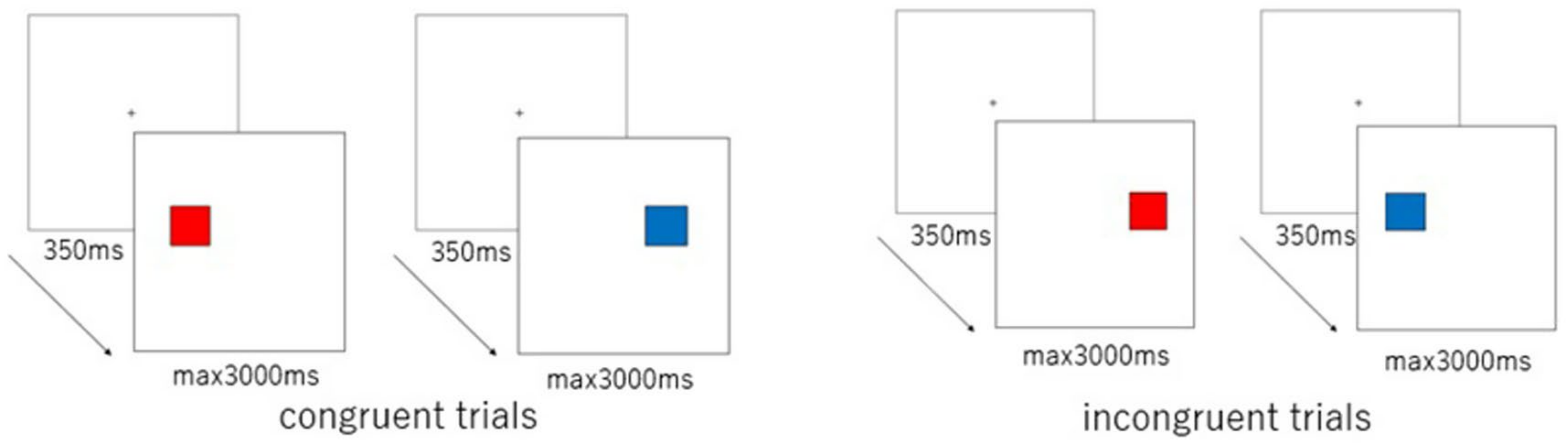
Stimulus presentation in congruent and incongruent trials for the Simon task.

The Simon task (Ross and Melinger, 2017; Figure 2) consisted of 48 trials. In 24 *congruent* trials, a red square appeared on the left side of the monitor, prompting the child to press the “S” key (marked with a red sticker) on the keyboard, or a blue square appeared on the right side, prompting the child to press the “M” key (marked with a blue sticker). The remaining 24 *incongruent* trials presented a red square on the right side, requiring the child to press the “S” key, or a blue square on the left side, requiring the child to press the “M” key. The sequence of stimulus presentation was automatically and pseudo-randomly determined by the experimental program. Responses and reaction times (RTs) were recorded. The Simon effect for each participant was calculated as the difference between the mean RT for correct responses (CRs) in incongruent trials and that in congruent trials.

#### DCCS task

**Figure 3:**
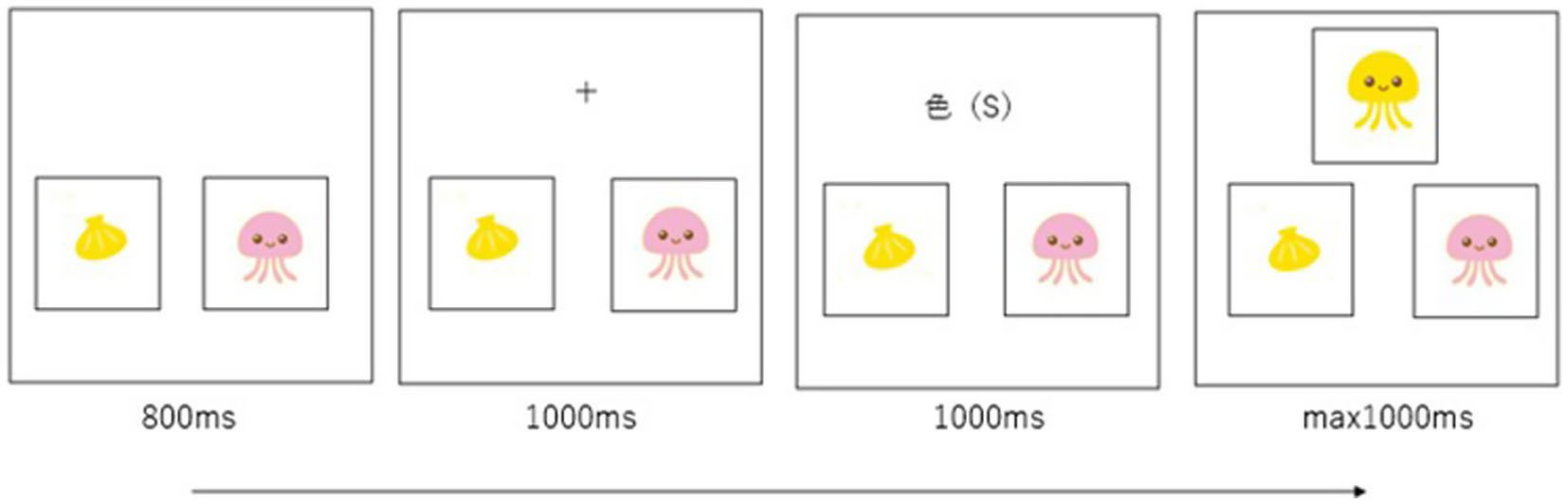
Stimulus presentation in the DCCS task. The cue word (color), where the child selected the drawing matching the sample in color, was “色(い)” (in the Japanese version) and “色(S)” (in the Chinese version). The cue word (shape), where the child selected the drawing matching the sample in shape, was “形(か)” (in the Japanese version) and “形(X)” (in the Chinese version).

The DCCS task, adapted from Zelazo et al. (2013) (Figure 3), consisted of three sequential sessions: color, shape, and mixed. After a training phase to familiarize the children with the procedure, they completed the three sessions in this fixed order.

In the *color session*, two drawings were displayed side by side in the lower half of the monitor. After the fixation point disappeared, the cue word “色(い)” (color) appeared at its location, instructing the child to focus on color. Immediately after the cue disappeared, a sample drawing was presented in the same location. The child was required to select the drawing that matched the sample in color by pressing the “←” key (for the left drawing) or the “→” key (for the right drawing) on the keyboard. This session consisted of five consecutive trials.

After Approximately 1 min, the *shape session* began, following the same procedure as the color session. However, the cue word “形(か)” (shape) appeared after the fixation point disappeared, prompting the child to focus on shape. The child was then asked to select the drawing that matched the sample in shape. Like the color session, this session also consisted of five consecutive trials.

After another 1-min interval, *mixed session* began. This session comprised 30 trials, including 24 shape trials (where the child selected the drawing matching the sample in shape) and 6 color trials (where the child selected the drawing matching the sample in color). The trials were automatically and pseudo-randomly presented by the experimental program. Responses and RTs were recorded for all sessions.

Two measures were derived from the DCCS task: (1) *the difference in CR ratios*, calculated as the ratio of CRs in shape trials minus that in color trials (“difference in CR in DCCS”), and (2) *the difference in RTs*, calculated as the mean RT for CRs in color trials minus that for CRs in shape trials (“difference in RT in DCCS”). These indices were computed for each participant.

#### Digit span task

The digit span task was administered following the procedure outlined in the *Wechsler Intelligence Scale for Children–Fourth Edition* (WISC-IV). In this task, children were required to repeat aloud the numbers they heard in reverse order. The auditory stimuli were recorded by a native Japanese speaker who spoke standard Japanese and by one of the authors, a native speaker of standard Chinese. After completing two practice trials, the test session began. The sequence of numbers started with three digits. If the child produced a CR on either the first or second attempt, the sequence length increased by one digit. Responses with no vocalization within a few seconds (approximately 3–5 s) were coded as “no response”; however, no child in the present sample met this criterion. If an incorrect response occurred on the first attempt, a different number sequence of the same length was presented for the second attempt. Each child’s score corresponded to the maximum sequence length they could accurately reproduce, ranging from three to nine. Two lists of digits sequences, each containing two different presentation patterns, were prepared and counterbalanced among participants.

#### Word reversal task

In the word reversal task, children were instructed to repeat aloud the words they heard in reverse order. The words ranged in length from two to four letters, and the number of words in each sequence presented varied from two to five. The auditory stimuli were the same as those used in the digit span task. After completing two practice trials, the test session began. In the first trial block, the child was presented with two words (e.g., *apple*, *dog*) and was required to repeat them in reverse order (*dog*, *apple*). If the child produced a CR on either the first or second attempt, the number of words in the next sequence was increased by one. The overall procedure was identical to that of the digit span task. Responses with no vocalization within a few seconds (approximately 3–5 s) were coded as “no response”; however, no child in our sample met this criterion. The procedure and scoring followed the method described by Ogawa and Koyasu (2008), with total scores ranging from two to five points. Two word lists, each containing words of equivalent length, were counterbalanced across participants. The words were carefully selected to ensure comparable difficulty and familiarity for young children, based on data from the National Institute for Japanese Language and Linguistics (1981). The selection process was conducted in collaboration with native Chinese- and Japanese-speaking psychologists.

#### 2.2.3 Boredom and concentration questionnaire

Parents completed a questionnaire assessing their children’s levels of boredom and concentration. The questionnaire included three items: one measuring the child’s state boredom during the experimental tasks (rated on a 0–8 scale), one assessing their daily tendency to experience boredom (boredom proneness; rated on a 0–8 scale), and one measuring their daily ability to concentrate (concentration proneness; rated on a 1–7 scale). The items assessing daily boredom tendency and daily concentration ability were identical to those used in Uehara and Ikegaya (2025), except that the response scale for boredom tendency was modified from 1–7 to 0–8 in the present study. The item assessing boredom state during the tasks was newly created for this study by adapting the wording of the daily boredom tendency item to the task context, with reference to Farmer and Sundberg (1986) single-item Likert measure of boredom state among university students during lectures. Additional questions unrelated to the present study’s aims were included as part of a separate project with different objectives and have not yet been published.

### 2.3 Indicator variables considered for analysis

This study examined the relationships between EF- related and boredom-related variables, as well as the effects of demographic variables on boredom. Three boredom-related indicators were analyzed: *state boredom*, *boredom proneness*, and *concentration proneness*. The EF-related indicators were categorized as follows.

Although the difference in the number of CRs between the color and shape sessions could serve as an indicator of cognitive flexibility, it was not included in the present analyses due to a ceiling effect, —approximately 70% of the children responded correctly in the shape session of the DCCS task. Consequently, four nonverbal EF indicators were used in the analyses:

1. The *dot matrix reversal task score* (“dot task score”), representing visual working memory;
2. The *Simon effect*, indicating response inhibition;
3. The *difference in CR in DCCS*, calculated as the ratio of CRs in shape trials minus that in color trials within the mixed session; and.
4. The *difference in RT in DCCS*, calculated as the mean RT for correct responses in color trials minus that in shape trials within the mixed session.

Both (3) and (4) reflect cognitive flexibility.

Additionally, two verbal EF indicators were included: the *digit span task score* and the *word reversal task score*, both assessing verbal working memory.

Multiple regression analyses were conducted to examine the extent to which boredom-related indicators were explained by EF-related and demographic variables (language group, gender, and age). The relationships between nonverbal EF and boredom-related indicators were analyzed using data from all participants. The relationships between Japanese verbal EF and boredom-related indicators were examined using data from Japanese monolingual and bilingual children, whereas those between Chinese verbal EF and boredom-related indicators were examined using data from Chinese monolingual and bilingual children.

Reaction time (RT) data were screened for distributional properties. Skewness and kurtosis values for the RT-based indices (the Simon effect and DCCS-related measures) indicated no substantial deviations from normality, and no extreme outliers were observed. Therefore, no RT data were excluded from the analyses.

### 2.4 Statistical analyses and use of large language models

All statistical analyses were performed using IBM SPSS Statistics, Version 29 (IBM Corp., Armonk, NY, USA). Because the sample size could not be determined *a priori* through power analysis, *post hoc* power analyses were conducted for each statistical test, and the corresponding power values are reported in the *Results* section. In the original manuscript, the language was reviewed by two professional editors who were native English speakers. For the revised manuscript, all English paraphrasing and grammatical refinements were performed with the assistance of ChatGPT (a large language model developed by OpenAI) to ensure clarity, consistency, and precision throughout the manuscript.

## 3 Results

### 3.1 Relationship between nonverbal EF variables and boredom-related variables

First, multiple regression analyses were conducted using nonverbal EF variables and demographic dummy variables as predictors for each of the three boredom-related indicators. The demographic variables included language group (Japanese monolingual vs. others, Chinese monolingual vs. others), gender, and age (6 years old vs. not, 7 years old vs. not). Across all three regression models, the variance inflation factor (VIF) for each of the nine predictors was below 2, indicating a very low risk of multicollinearity (Kutner et al., 2004) (Table 1). In addition, the Durbin–Watson statistic was close to 2, suggesting that the residuals were randomly distributed.

**Table 1:** Coefficients for “group (JP monolingual or not),” “gender,” “age (6 years or not),” “age (7 years or not),” “Dot task score,” “Simon effect,” “Difference in CR in DCCS,” and “Difference in RT in DCCS” in multiple regression analyses and the VIF.

Dependent variable	Effect	*B*	SE	*β*	*t*	*P*	VIF
State boredom (*n* = 84)	Intercept	2.946	1.425		2.068	0.042*	
	**Group (JP monolingual or not)**	**0.940**	**0.542**	**0.213**	**1.736**	**0.087** ^**†**^	**1.459**
	Group (CH monolingual or not)	−0.365	0.565	−0.083	−0.647	0.520	1.586
	**Gender (Boy or not)**	**1.432**	**0.452**	**0.341**	**3.169**	0.002**	**1.123**
	Age (6 years or not)	−0.791	0.607	−0.182	−1.304	0.196	1.887
	Age (7 years or not)	−0.446	0.582	−0.101	−0.767	0.446	1.688
	Dot task score	−0.056	0.061	−0.097	−0.916	0.363	1.097
	Simon effect	−2.718	2.877	−0.107	−0.945	0.348	1.237
	Difference in CR in DCCS	−1.375	1.089	−0.143	−1.262	0.211	1.247
	Difference in RT in DCCS	0.121	0.515	0.025	0.235	0.815	1.058
Boredom proneness (*n* = 86)	Intercept	4.566	1.476		3.094	0.003**	
	Group (JP monolingual or not)	0.824	0.573	0.179	1.437	0.155	1.476
	Group (CH monolingual or not)	0.035	0.594	0.008	0.059	0.953	1.633
	Gender (Boy or not)	0.770	0.471	0.177	1.637	0.106	1.112
	Age (6 years or not)	−0.576	0.633	−0.127	−0.910	0.366	1.856
	Age (7 year or not)	−0.507	0.602	−0.111	−0.843	0.402	1.627
	Dot task score	−0.094	0.063	−0.158	−1.480	0.143	1.083
	Simon effect	−1.07	2.978	−0.041	−0.359	0.720	1.219
	**Difference in CR in DCCS**	**2.292**	**1.143**	**0.228**	**2.006**	0.048*	**1.225**
	**Difference in RT in DCCS**	**0.959**	**0.541**	**0.187**	**1.772**	**0.080** ^**†**^	**1.051**

Bold values represent statistically significant or marginally significant effects, except for intercept.

**p < 0.01; *p < 0.05; ^†^*p* < 0.1. VIF, variance inflation factor.

The regression model with state boredom as the dependent variable accounted for 23.7% of the variance [*F* (9,74) = 2.55, *p* = 0.013, adj. *R*^2^ = 0.144, power = 0.690]. As shown in Table 1, gender (boy vs. not) was significantly and positively associated with state boredom (*p* = 0.002), and language group (Japanese monolingual vs. others) showed a marginally significant positive association (*p* = 0.087). These findings suggest that boys tended to experience greater boredom during EF tasks than girls, and that Japanese monolinguals reported slightly higher boredom levels than Chinese monolinguals and bilinguals.

The regression model with boredom proneness as the dependent variable explained 19.7% of the variance [*F* (9,76) = 2.07, *p* = 0.042, adj. *R*^2^ = 0.102, power = 0.498]. As shown in Table 1, the “difference in CR in DCCS” was significantly and positively associated with boredom proneness (*p* = 0.048), and the “difference in RT in DCCS” showed a marginally significant positive association (*p* = 0.080). These results indicate that lower cognitive flexibility—reflected by larger differences in CR and RT in the DCCS—is associated with higher boredom proneness.

Finally, the multiple regression model with concentration proneness as the dependent variable was judged to have low validity, as the overall *R*^2^ was small and the *F*-test was not statistically significant [*R*^2^ = 0.160, *F* (9,76) = 1.61, *p* = 0.129].

### 3.2 Relationships between Japanese verbal EF variables and boredom-related variables

Multiple regression analyses were conducted to examine the associations between Japanese verbal EF variables (digit span and word reversal task scores) and the three boredom-related indicators, controlling for demographic variables [language group (Japanese monolingual vs. bilingual), gender (boy vs. not), and age (6 years old vs. not)]. The variable “age (7 years old vs. not)” was excluded from all three models because its inclusion produced in VIFs greater than 2 for both age variables.

Across all models, the VIF values for the five predictors were below 2, and the Durbin–Watson statistics were close to 2, indicating minimal multicollinearity and no evidence of autocorrelation.

The regression models with state boredom and boredom proneness as dependent variables showed low explanatory power, with low *R*^2^ values and non-significant *F*-tests [state boredom: *R*^2^ = 0.113, *F* (5,51) = 1.31, *p* = 0.227; boredom proneness: *R*^2^ = 0.159, *F* (5,51) = 1.92, *p* = 0.107]. Conversely, the model with concentration proneness as the dependent variable explained 18.5% of the variance and was marginally significant [*F* (5,51)= 2.31, *p* = 0.057, adj. *R*^2^ = 0.105, power = 0.434].

As shown in Table 2, concentration proneness was significantly and negatively associated with gender (boy vs. not) (*p* = 0.042) and significantly and positively associated with Japanese word reversal task score (*p* = 0.017). These findings suggest that girls and individuals with higher scores on the Japanese word reversal task tend to exhibit greater concentration ability in daily life.

**Table 2:** Coefficients for “group,” “gender,” “age (6 years or not),” “digit span,” and “word reversal” in multiple regression analyses and the VIF.

Language and dependent variables	Effect	*B*	SE	*β*	*t*	*P*	VIF
Japanese concentration proneness (*n* = 57)	Intercept	5.120	1.078		4.749	0.000**	
	Group (JP monolingual or not)	−0.247	0.335	−0.097	−0.736	0.465	1.096
	**Gender (Boy or not)**	−**0.672**	**0.322**	−**0.265**	−**2.090**	0.042*	**1.009**
	Age (6 years or not)	−0.343	0.347	−0.136	−0.986	0.329	1.186
	Digit span	−0.272	0.236	−0.178	−1.155	0.253	1.479
	**Word reversal**	**0.661**	**0.269**	**0.349**	**2.457**	0.017*	**1.262**
Chinese state boredom (*n* = 56)	Intercept	2.368	1.216		1.946	0.057^†^	
	Group (CH monolingual or not)	−0.231	0.555	−0.057	−0.417	0.678	1.227
	**Gender (Boy or not)**	**1.494**	**0.510**	**0.366**	**2.928**	0.005**	**1.031**
	**Age (6 years or not)**	−**1.145**	**0.627**	−**0.245**	−**1.825**	**0.074** ^**†**^	**1.182**
	Digit span	0.242	0.217	0.164	1.112	0.272	1.435
	Word reversal	−0.610	0.376	−0.247	−1.621	0.111	1.532

Bold values represent statistically significant or marginally significant effects, except for intercept.

**p < 0.01; *p < 0.05; ^†^*p* < 0.1. VIF, variance inflation factor.

### 3.3 Relationship between Chinese verbal EF variables and boredom-related variables

Multiple regression analyses were conducted to examine the associations between Chinese verbal EF variables and the three boredom-related indicators, controlling for demographic variables (language group [Chinese monolingual vs. bilingual], gender [boy vs. not], and age [6 years old vs. not]). The variable “age (7 years old vs. not)” was excluded from all three models because its inclusion resulted in lower *F*-values and adjusted R^2^ values.

Across all models, the VIF values for the five predictors were below 2, and the Durbin–Watson statistics were close to 2, indicating minimal multicollinearity and no evidence of autocorrelation.

The regression models with boredom proneness and concentration proneness as dependent variables showed low explanatory power, with low *R*^2^ values and non-significant *F*-tests [boredom proneness: *R*^2^ = 0.081, *F* (5,52) = 0.91, *p* = 0.479; concentration proneness: *R*^2^ = 0.144, *F* (5,52) = 1.74, *p* = 0.141]. In contrast, the model with state boredom as the dependent variable explained 24.0% of the variance and was statistically significant [*F* (5,50) = 3.16, *p* = 0.015, adj. *R*^2^ = 0.164, power = 0.670].

As shown in Table 2, state boredom was significantly and positively associated with gender (boy vs. not) (*p* = 0.005) and negatively associated with age (6 years old vs. not) (*p* = 0.074). These findings suggest that boys tend to experience higher levels of boredom during EF tasks than girls, and that six-year-olds are less likely to experience boredom compared with seven- and eight-year-olds.

## 4 Discussion

This study yielded three primary findings. First, in the analyses involving nonverbal EF variables, being a boy was associated with greater boredom during EF tasks. In addition, Japanese monolingual children tended to report slightly higher boredom levels compared with other language groups. Moreover, cognitive flexibility was inversely related to dispositional boredom proneness, indicating that lower cognitive flexibility was associated with greater susceptibility to boredom. Second, in the analyses of Japanese verbal EF variables, being a girl and achieving higher scores on the Japanese word-reversal task were both linked to a greater ability to concentrate in daily life. Third, regarding Chinese verbal EF variables, boys were again more prone to boredom during tasks, whereas six-year-olds were less likely to experience boredom compared with older peers.

With respect to state boredom, no significant associations were observed with either nonverbal or verbal EF. However, several demographic factors were significantly related to state boredom: being a boy—and, to a lesser extent, being older than six years or being a Japanese monolingual—was associated with higher boredom levels. Cognitive flexibility was significantly related to boredom proneness, and verbal working memory was significantly associated with concentration ability. Given that the ease of concentration can be viewed as the inverse of boredom (Farmer and Sundberg, 1986), these results suggest that both cognitive flexibility and verbal working memory are linked to individual differences in tendencies toward boredom and concentration.

The gender differences observed in state boredom were not unexpected, as previous studies have reported higher boredom levels among men (e.g., Sundberg et al., 1991; Vodanovich et al., 2011; Wang et al., 2022). However, studies conducted in Japan have found no significant gender differences in children’s everyday boredom proneness (Uehara and Ikegaya, 2025) or in adults’ Boredom Proneness Scale scores (Uehara et al., 2024). Because gender differences in boredom-related measures have not been consistent across studies, further research is needed to clarify which aspects of boredom show gender differences and how these differences may change across development. In addition, previous findings have suggested that state boredom tends to increase with age in adolescents as attention span develops (Perone et al., 2023). The partial age differences in state boredom observed in this study may therefore reflect developmental changes in the experience of boredom, which also warrants further investigation. Linguistic and cultural differences were also partially evident in the present findings. Japanese monolingual children showed significantly higher levels of state boredom, whereas among Japanese-speaking children (both monolingual and bilingual), higher verbal working memory scores tended to be associated with a greater tendency toward concentration. The former pattern suggests group differences in state boredom during the task, rather than differences in trait-level boredom proneness, whereas the latter pattern was not observed among the Chinese-speaking group. These results may partly reflect parental rating bias among the Japanese monolingual group—specifically, parents who themselves had been educated in Japan might have perceived the EF tasks used in this study, which resembled schoolwork, as inherently boring. Consequently, they may have rated their children’s state boredom higher than parents in other groups. Alternatively, this group difference could simply be due to chance, given the relatively small sample sizes for each group. Further research is needed to determine whether these linguistic and cultural differences stem from language-related developmental processes or from broader cultural factors.

Among the EF domains, inhibition, as assessed by the Simon task, was not associated with dispositional boredom or concentration proneness. Visual working memory, reflected in the dot matrix reversal task and the digit span task, was also unrelated to these variables. In contrast, both cognitive flexibility and verbal working memory, as measured by the word reversal task, were implicated. The difference between the word reversal and digit span tasks—although both engage verbal working memory to some extent—likely lies in the degree to which they rely on lexical knowledge and academic verbal skills. This distinction may account for the differential results observed among EF components, particularly considering that extreme boredom can often be self-regulated. Further investigation of strategies to manage boredom effectively may have important implications, especially for children with attention deficit–hyperactivity disorder, who frequently exhibit EF dysfunction characterized by difficulties in emotion regulation (Groves et al., 2022) and heightened boredom proneness (Golubchik et al., 2020; Malkovsky et al., 2012). A shared underlying mechanism is also possible, as Poon et al. (2022) reported that cognitive flexibility and working memory mediate the relationship between socioeconomic status and academic performance. These findings are consistent with the present results, given that verbal working memory—reflected in the word reversal task—is closely linked to verbal academic learning. This is also in line with studies showing associations between state boredom in children and academic achievement (Jie et al., 2022; Tze et al., 2016). Thus, fostering self-regulation skills, including cognitive flexibility and working memory, may help children manage boredom more effectively, thereby supporting performance across multiple domains, including academics. Nonetheless, further research is warranted, as the significant association between verbal working memory and concentration was not observed among Chinese monolinguals.

This exploratory study has several limitations. Although the EF data were derived from children’s performance on various experimental tasks, the boredom-related data were based solely on parent ratings obtained via three Likert scales. Given the lack of widely established measures for assessing children’s boredom, identifying alternative, more accurate methods was challenging. Future research should investigate more direct approaches to measuring boredom in children. Nevertheless, the validity of the current approach is supported by several adult studies that have reported significant correlations between boredom proneness scales and Likert-type ratings assessing boredom proneness and concentration tendencies (e.g., Farmer and Sundberg, 1986; Uehara et al., 2024). Furthermore, the emotional dimension of boredom warrants closer consideration, and future studies should directly examine how children regulate emotions in situations eliciting boredom. Although intelligence was not assessed in the present study, it may be partially or indirectly related to both boredom and executive functioning, particularly in childhood; thus, future research should consider intelligence as a potentially important factor. Additional limitations should also be acknowledged. Our assessment focused on a limited subset of executive functions, and other unmeasured variables—including potential cultural and contextual differences between Japan and China— may have influenced the results. The range of bilingual participants (and, by extension, cultural backgrounds) was restricted, and the operational definition and measurement of bilingualism were relatively broad. To enhance the validity and generalizability of future findings, subsequent studies should recruit larger and more diverse samples that span broader age ranges, cultural contexts, and bilingual proficiency levels, and should examine multiple aspects of cognitive functioning, including executive functions. Moreover, because the current assessments relied exclusively on parental reports, future research should incorporate children’s self-reports or teacher evaluations to obtain a more balanced understanding of children’s emotional and behavioral characteristics. Finally, we were unable to conduct an *a priori* power analysis; instead, *post hoc* power analyses were performed for each test, and the results are reported in the Results section. Although two of the regression models demonstrated acceptable power values (approximately 0.7), the other two models yielded relatively low power. Consequently, the present findings should be interpreted as exploratory, and further data collection with larger samples will be necessary to validate these results.

In conclusion, by incorporating multiple EF tasks, this study was the first to demonstrate that specific components of executive function—particularly cognitive flexibility and verbal working memory—are more closely associated with children’s everyday boredom proneness than with their momentary state boredom during tasks. These findings provide valuable insight into how self-regulatory capacities may shape boredom tendencies in childhood.

## Data Availability

The raw data supporting the conclusions of this article will be made available by the authors, without undue reservation.
